# Supplementary material for: Retinal photoisomerization versus counterion protonation in light and dark-adapted bacteriorhodopsin and its primary photoproduct
Source: Nat Commun. 2024 Mar 8;15:2136. doi: 10.1038/s41467-024-46061-w (PMC10923925; doi:10.1038/s41467-024-46061-w)
Supplement: Supplementary file 1 — Supplementary Information [file 41467_2024_46061_MOESM1_ESM.pdf]

Supplementary Information

Retinal photoisomerization vs counterion protonation in light and dark-adapted bacteriorhodopsin and its primary photoproduct

Partha Malakar[a‡], Samira Gholami[b‡], Mohammad Aarabi[b], Ivan Rivalta[b,c], Mordechai Sheves*[d], Marco Garavelli*[b] and Sanford Ruhman*[a]

[a] Institute of Chemistry, The Hebrew University of Jerusalem, Jerusalem, 9190401, Israel

[b] Dipartimento di Chimica industriale "Toso Montanari", Università di Bologna, Viale del Risorgimento 4, 40136 Bologna, Italy

[c] ENSL, CNRS, Laboratoire de Chimie UMR 5182, 46 allée d'Italie, 69364 Lyon France

[d] Department of Molecular Chemistry and Materials Science, The Weizmann Institute of Science, Rehovot 7610001, Israel

[‡]These authors equally contributed to the work

Table of Contents:

Supplementary Note 1: Computational details:

1.1 Computational Spectroscopy

Supplementary Note 2: Photochemistry of LA- and DA-BR

2.1 530 nm pump pulse characterization

2.2 TA data of DA-BR

2.3 Isolation of 13C photochemistry

2.4 Isomer ratio determination for DA-BR

2.5 Quantum efficiency of 13-*cis* 15-*syn* (13C) to all-*trans* 15-*syn* (K') photoisomerization

2.6 Computational results

Supplementary Note 3: Photochemistry of K intermediate

3.1 640 nm pump pulse characterization

3.2 Quantification of K contribution in the three-pulse measurement

3.3 Computational results

Supplementary references

Supplementary Note 1: Computational details

Supplementary Fig. 1 shows the general scheme of the bacteriorhodopsin (hereafter BR) QM/MM model constructed by Protocol-1 described in the method section of the main text.

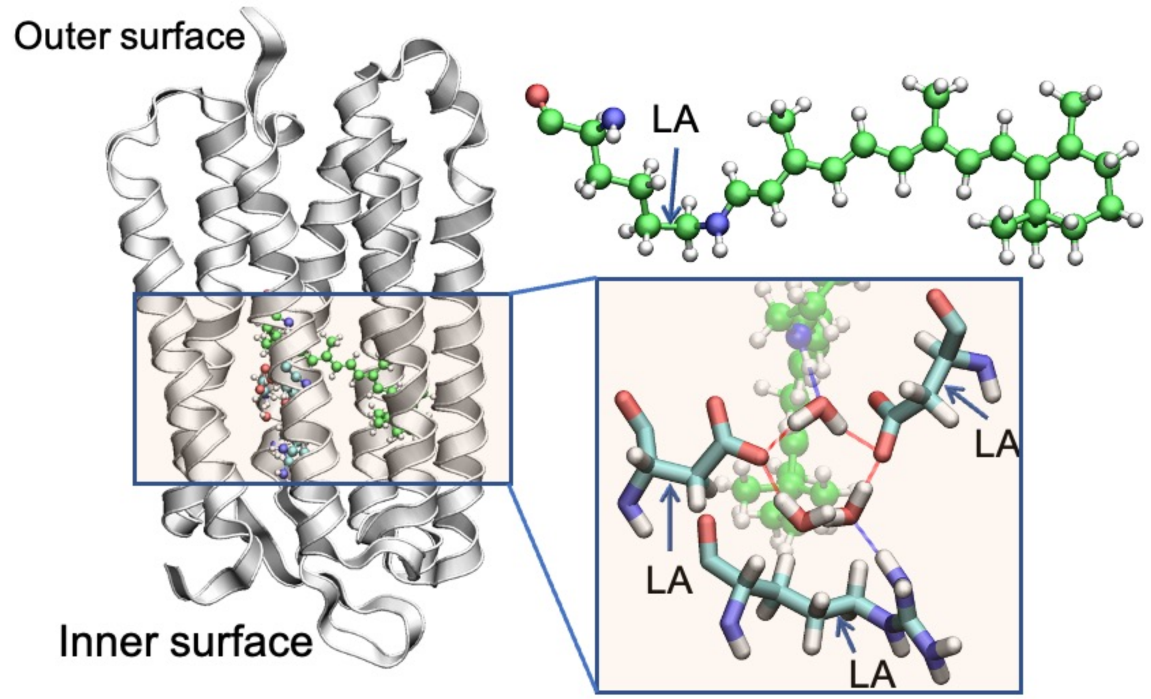

Supplementary Figure 1. General scheme of bR QM/MM model of the Protocol-1. This is composed by: (left) environment subsystem (gray cartoon), chromophore rPSB and LYS-216 side-chain covalently connected to the chromophore (green ball-and-sticks), ASP85, ASP212, ARG82 and three crystallographic waters participating in the H-bond network (cyan ball-and-sticks) of the quadrupole in the standard protonation state. Right: QM region contains the rPSB chromophore and the linked LYS-216 side chain fragment were cut between Cε−Cδ, sidechains of the ASP85, ASP212 and ARG82 were cut between Cα and Cβ and three crystallographic waters. The H-link atom has been indicated with "LA" in the inset.

1.1 Computational Spectroscopy

Throughout this work electronic (LA) and vibrational (IR) spectroscopy has been used to validate the constructed models of three BR states and assign the correct protonation state of the quadrupole in AT state, respectively. Accordingly, the excited-state electronic structure (i.e. vertical

excitation), normal modes, and associated frequencies have been computed on top of the optimized geometries of all interested systems.

IR spectroscopy: In order to assess the reliability of the various (fully deprotonated or singly protonated aspartate) models of all-*trans* BR (AT-BR), we have simulated their IR spectra by means of density functional theory (DFT) accounting for a QM description of the entire complex counterion within a QM/MM scheme. Specifically, all vibrational frequency calculations in the ground state of AT-BR in five different protonation states, were performed at B3LYP/6-31G(d)/MM level of theory. The optimized models obtained by Protocol-1 were used for QM/MM computations of IR spectra. The QM/MM frequencies were computed numerically with COBRAMM package[1] by displacing only high layer (i.e. QM; Supplementary Fig. 1) atoms. A uniform scaling factor of 0.97 was applied as suggested by the literature. The scaling was chosen so to calibrate the frequency of the IR vibrations as obtained from QM/MM computations to their experimental values. Spectra are convoluted with Lorentzian with a broadening of 10 cm^{-1} and are shown over the range 2000–3600 cm^{-1}.

As shown in Supplementary Fig. 10 A-C, the most intense IR vibrational modes computed for the standard complex counterion feature various combinations of O–H and N–H stretching's, indicating a certain degree of complexity that is associated with the interpretation of previous experimental data. Still, two of the main modes described above, i.e. ν_{NH} and ν_1, are found as intense peaks in the computed IR spectrum of the standard complex counterion but their frequencies are largely red-shifted with respect to the experimental values, with ν_1 at 2061 cm^{-1} (instead of 3000 cm^{-1}) and ν_{NH} at 2638 cm^{-1} (instead of 2750-2800 cm^{-1}). The ν_2 mode associated to the H-bond between negative ASP85 and w401, instead, is found at the appropriate frequency range (3055 instead 3100 cm^{-1}), but is largely mixed with the O–H stretching of w402, also H-bonded to ASP85. These results indicate that the doubly deprotonated model of the complex counterion is not fully consistent with the available experimental IR data.

By protonating ASP212, see Supplementary Fig. 10 D-E, the computed IR modes maintain the mixing between the stretching modes observed in the standard model but they feature vibrational frequencies better matching with experiments. Independently on the carboxylic oxygen protonated, with the proton added to ASP212 being part or not of the H-bonding network of the complex

counterion, these models present modes that are quite extended along the H-bonding network connecting the N–H of PSB with that of ARG82, (see Supplementary Fig. 10 D). Notably, the protonated ASP212 model features IR spectra that are mostly consistent with the experimental observation of two bands, one around $\simeq$2800 cm^{-1} with participation of PSB and w402 and one $\simeq$3000 cm^{-1} with involvement of w401, w406 and ARG82.

The first consequence of the protonation of ASP85 (see Supplementary Fig. 10 F-G) is, unsurprisingly, the disappearance of the ν_2 mode associated to the strong H-bond between negative ASP85 and w401. Moreover, the ν_{NH} band at $\simeq$2800 cm^{-1} is largely blue-shifted to $\geq$3000 cm^{-1}, as a consequence of the H-bond with the bridging w402, which now is strongly H-bonded to negative ASP212 and weakly interacting with ASP85. Overall, the doubly deprotonated (standard) and the protonated ASP85 models have much worse agreement with experiments than protonated ASP212 models, suggesting the latest being the favored protonated site for the complex counterion. Remarkably, this is consistent with ASP85 acting as the primary acceptor for retinal deprotonation, as widely recognized and consolidated.[2–4]

LA spectroscopy*:* We relaxed the rPSB at CASSCF/MM level of theory, keeping the environment (MM part) frozen in its DFT optimized position (Protocol-2). Energies have been corrected by means of multi-reference second order perturbations, at single state (SS) CASPT2/MM level of theory. Avoiding so-called "intruder states" has been done by using an imaginary shift of 0.2, and no IPEA has been set. Oscillator strengths have been evaluated using CASPT2 energies with the RASSI program as implemented in Molcas 8.[5] The 6-31G(d) basis set has been used for all the computations.

Supplementary Note 2: Photochemistry of LA- and DA-BR

2.1 530 nm pump pulse characterization

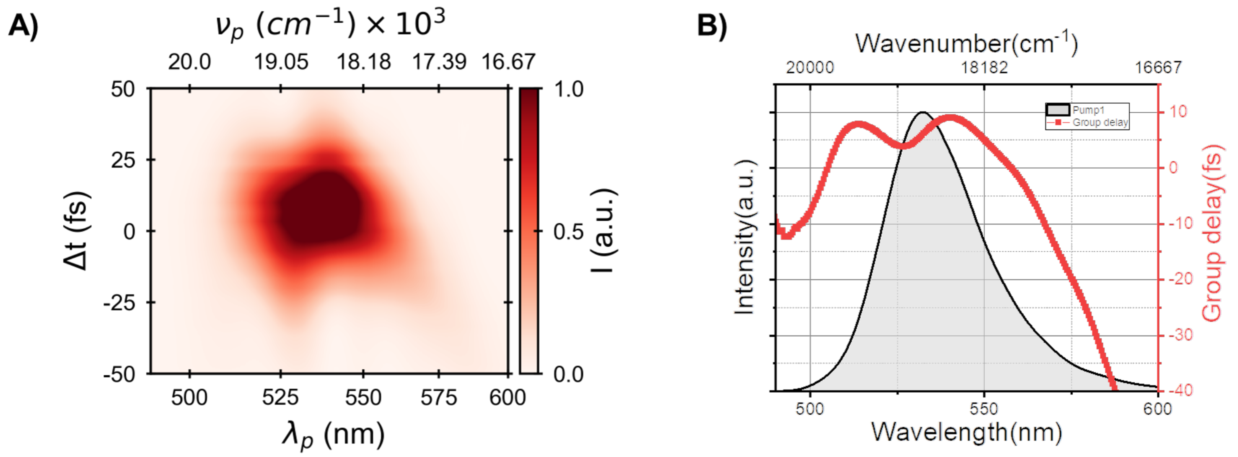

Supplementary Figure 2. Pump1 spectra. A) 2D colour map of FROG for the Pump1. X-axis represents wavelength, y-axis represents the delay and transmitted intensity is colour coded. B) The pump1 spectrum and group delay obtained from FROG. Left y-axis stands for normalized pump intensity and right y-axis (red) represents group delay. Source data are provided as a Source Data file.

2.2 TA data of DA-BR

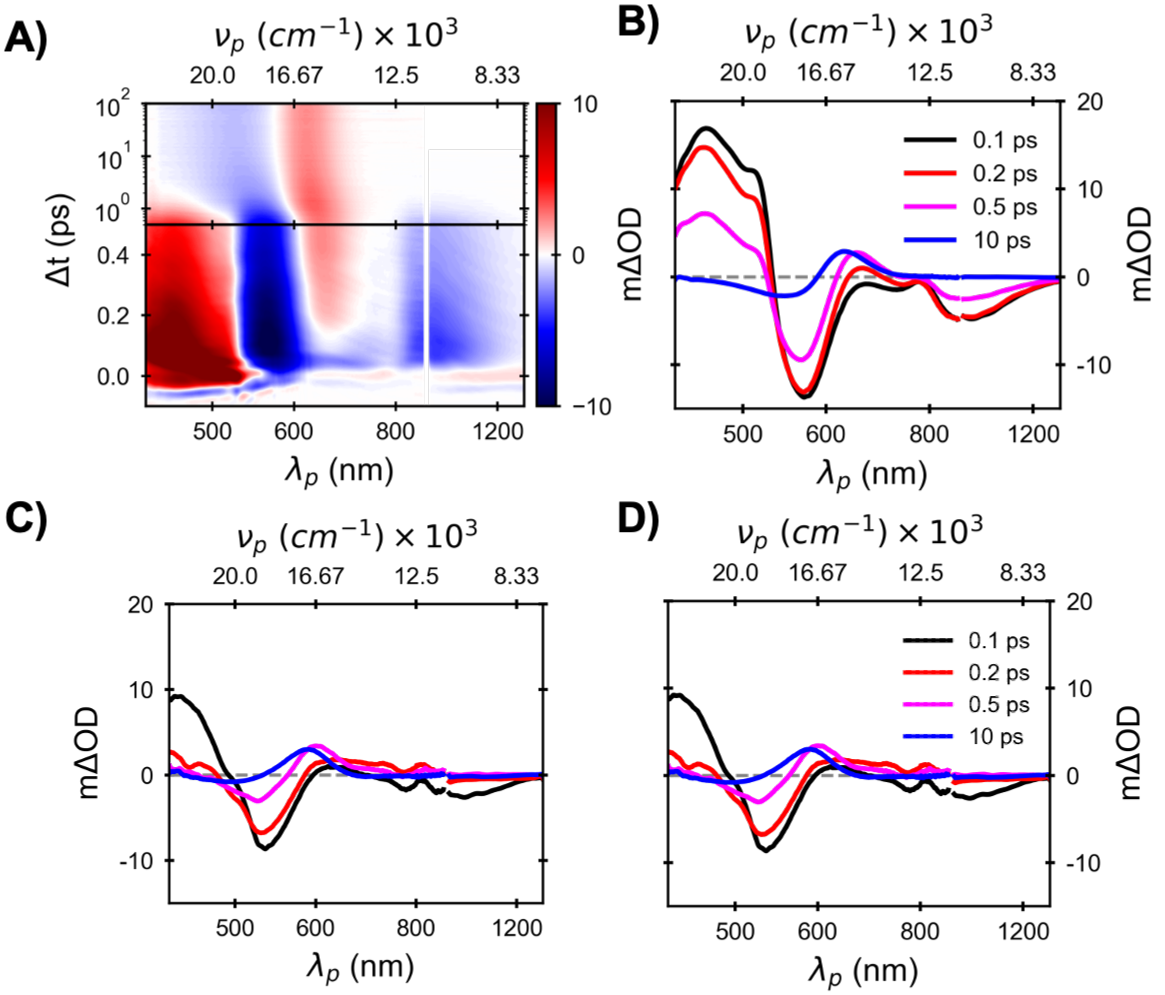

Supplementary Figure 3. TA data. A) The 2D colormap of TA data of DA-BR. X-axis represents probe wavelength or wavenumber. Y-axis represents the delay between pump and probe. Initial 500 fs pump-probe delay is linear, and rest of the axis is logarithmic. The absorption difference is color coded: color bar at the right of the map presents the value of absorption difference. TA Spectra at various pump-probe delay for B) DA, C) LA and D) 13C. Source data are provided as a Source Data file.

2.3 Isolation of 13C photochemistry

Previous estimates of isomer ratio suggest DABR contains about 60 % 13C and 40 % AT. As the DABR is excited at the isosbestic point between 13C and AT absorption, 13C photochemistry could be extracted by subtracting 40 % AT component. Based on this ratio subtracted 13C stimulated emission TA data contains a very fast component and a 500-fs decay component. But 500 fs decay component is due to AT. Therefore, based on this ratio subtraction leads to erroneous result.

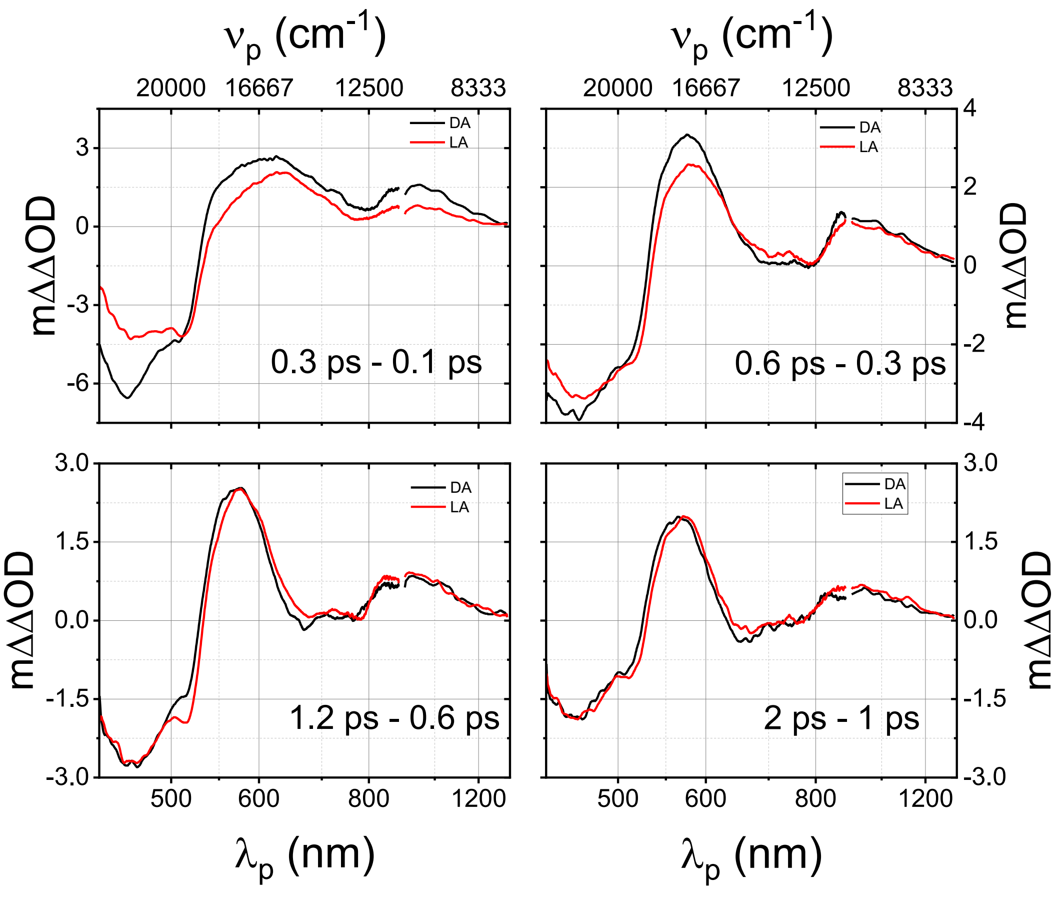

Supplementary Figure 4. Dynamic difference spectra. Dynamic difference spectra [ΔΔOD =ΔOD(t+δt)-ΔOD(t)], at various stages of excited state decay. The relevant time information is provided in each graph. Source data are provided as a Source Data file.

2.4 Isomer ratio determination for DA-BR:

The scaling factor obtained from dynamic difference is 0.67, which indicates a ratio of 13C and AT in the DA sample is 35:65 that differs significantly from the literature value of 60:40 to at most 50:50.[6] We did another dedicated measurement to answer the ambiguity of the isomer ratio. It is evident from previous sections that the 13C excited state activity is limited to 200 fs. Therefore, the stimulated emission of DA sample after 200 fs is solely coming from AT. Thus, after a few hundred femtoseconds, the SE value could measure AT contribution. The value of stimulated emission must reduce from LA to DA, and the ratio of SE signature between DA and LA is the AT fraction in the DA-BR. Hence, we carried out TA measurements for LA and DA samples with a narrow band pump at 500 nm, resulting fraction is 0.65 ± 0.03 (Supplementary Fig. 5 A, B, C). Next, we repeated the measurement by changing the excitation to 535, 560, and 610 nm (Supplementary Fig. 5 A, B, D, E, F). The fraction remained identical despite the excitation wavelength variation (Supplementary Fig. 5 B). Therefore, the ratio between 13C and AT in the DA-BR is 35:65.

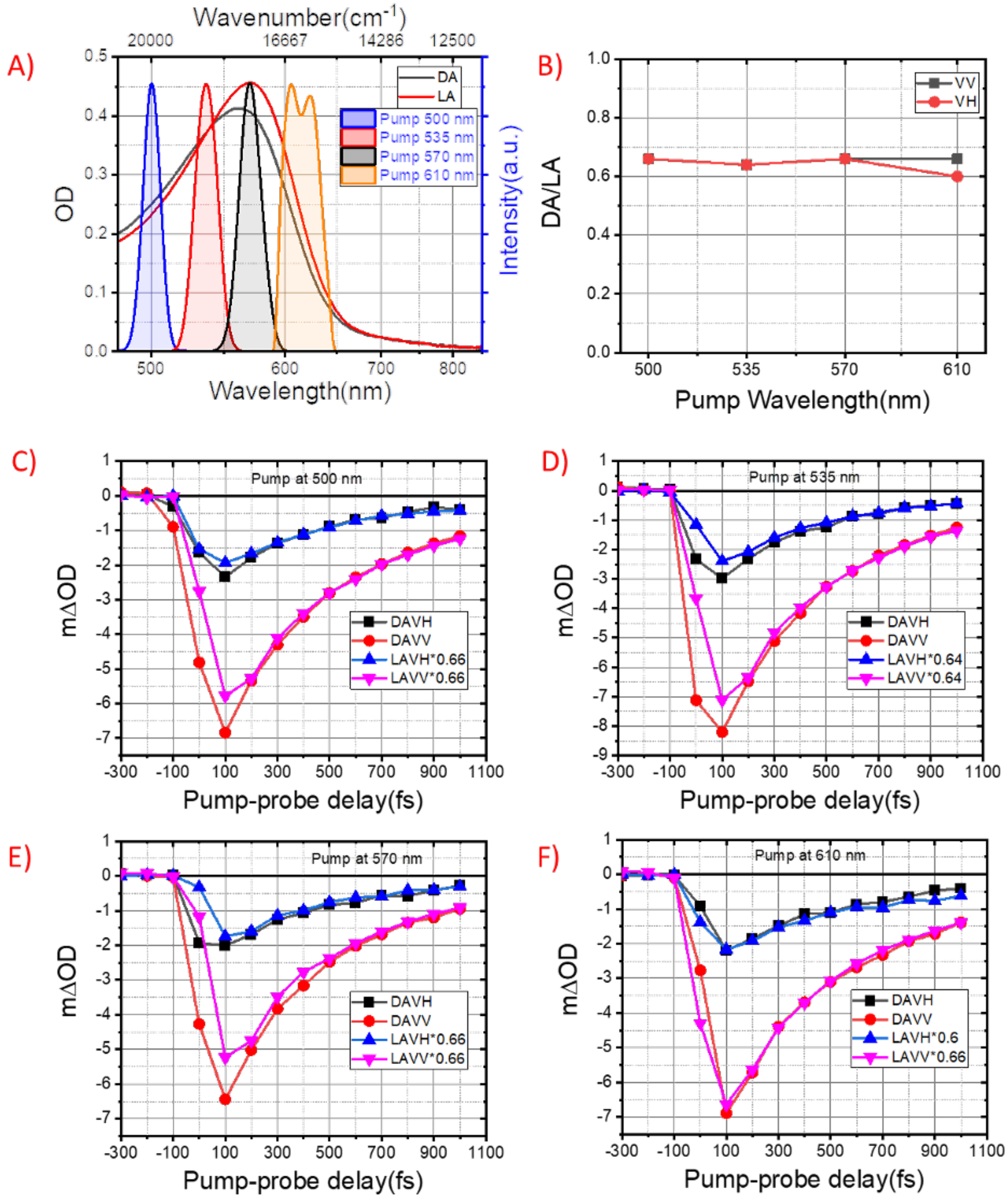

Supplementary Figure 5. TA measurements with narrow band pumps. A) *Light and dark-adapted bacteriorhodopsin absorption spectra and narrow band pump spectra. B) Ratio of AT component between DA and LA-BR. C)-F) Stimulated emission decay of DA-BR and scaled LA-BR for various excitation. Identical pump and probe polarization (VV); Orthogonal polarization between pump and probe (VH). Source data are provided as a Source Data file.*

2.5 Quantum efficiency of 13-*cis* 15-*syn* (13C) to all-*trans* 15-*syn* (K') photoisomerization:

13C resting state always accompanies AT resting state, so measuring 13C to AT photoisomerization quantum efficiency remains challenging. AT isomer has a 1.18 times higher transition dipole than 13C, irrespective of the 15 positions.[7] As in the case of BR identical bandwidth integrated area for AT is 2.3×10^8 , 13C is 1.94×10^8, and K is 1.96×10^8 L mol^{-1} cm^{-2} (Supplementary Fig. 6).

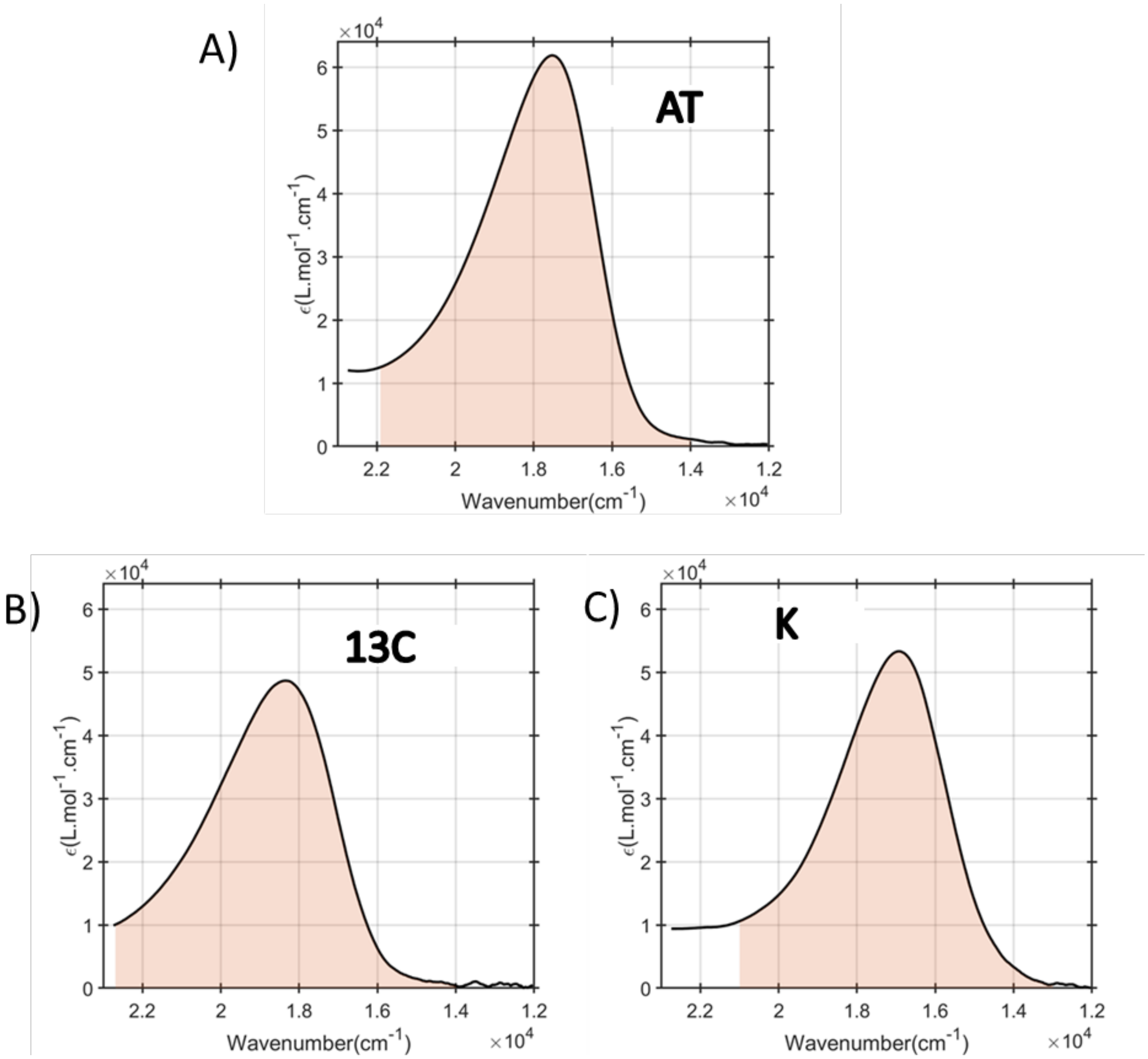

Supplementary Figure 6. Absorption spectrum of BR isomers. Absorption spectrum of BR isomers is presented as a black line, and the integrated area are highlighted. Source data are provided as a Source Data file.

Since our TA spectrum at a longer pump-probe delay (> 30 ps) is absorption different between the isomers and individual isomers have their characteristic absorption frequency, we cannot use the identical width integrated area value. Instead of identical width integration, we use constant range integration. The constant range integration values of individual isomers and differences are given in Supplementary Table 1. Noticeably, the AT - 13C integrated area is almost double AT-K at this constant frequency range. Therefore, if AT - 13C integrated area is 1.7 times of AT-K the quantum efficiency of isomerization starting from 13C is 64 %.

Supplementary Table 1: Integrated area parameters.

	Wavenumber range (cm^{-1})	Integrated area ($\times 10^8$ L mol^{-1} cm^{-2})
AT	13331 - 22233	2.33
13C	13331 - 22233	1.90
K	13331 - 22233	2.08
AT - 13C	13331 - 22233	0.43
AT - K	13331 - 22233	0.25

Our TA data of the DA sample contains ~35 % 13C isomer and ~65 % all-trans isomer. Since we have excited around the isosbestic point, dynamic difference spectra also reflected a

35:65 ratio between 13-*cis* isomer and all-trans isomer. Thus, our subtracted 13C TA data is for 35% of 13C concentration. We could easily scale 13C data for 100%. The identical concentration data of 13-*cis* and AT is used for the next section.

The integrated TA spectra of the constant range for AT and 13C for entire pump-probe delay are presented in Supplementary Fig. 7A. The integrated area of TA spectra starting from the 13C photocycle is twice of AT photocycle. It suggests that 13C to K' isomerization quantum efficiency is 70 ± 20 %. The difference spectra between DA-LA and the late time TA spectra of 13-*cis* photocycle have remarkable similarities (Supplementary Fig. 7B). Suggesting all-*trans* 15-*syn* intermediate (K') starting from 13C and all-*trans* 15-*anti* resting state (AT or LA) have a similar absorption spectrum.

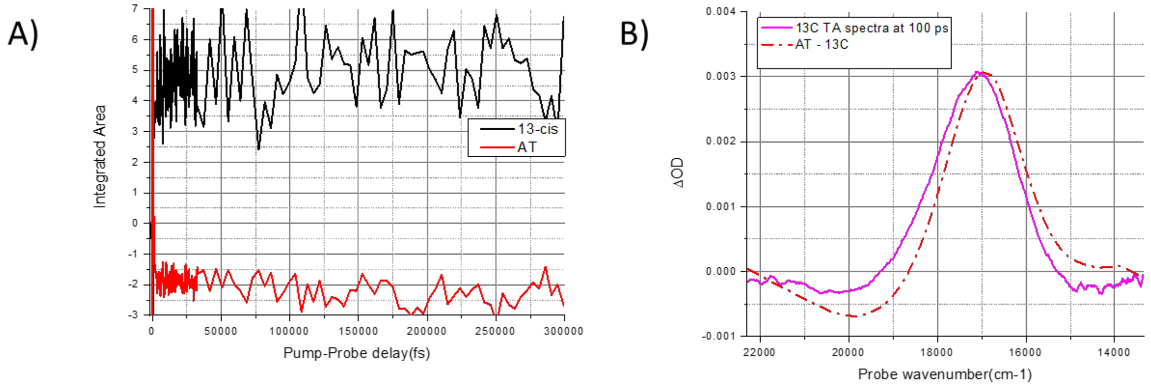

Supplementary Figure 7. TA spectra and integrated area. A) Integrated area of TA spectra. A fixed probe wavenumber range (13331-22233 cm^{-1}) is used to evaluate the integrated spectrum (AT=2.5 ± 0.5 unit and 13C = 5 ± 0.1 unit). B) TA spectra at 100 ps for 13C and AT-13C absorption difference. Source data are provided as a Source Data file.

2.6 Computational results

In order to identify the molecular origin of the different dynamical behavior (ballistic *vs* non-ballistic) of BR in different states (DA *vs* LA) we have computed the potential energy surface

along the BLA and minimum energy photoisomerization paths (MEPs) along the dihedral angle, C_{12}–C_{13}=C_{14}–C_{15}, as described in the Method section in the main text. The resulting structures from Protocol-2 (see Method section in the main text), has been used to build these energy profiles. Supplementary Fig. 8 shows the BLA relaxation profile and photoisomerization MEPs of LA and DA states of BR for the standard counterion model (fully deprotonated ASP85 and ASP212).

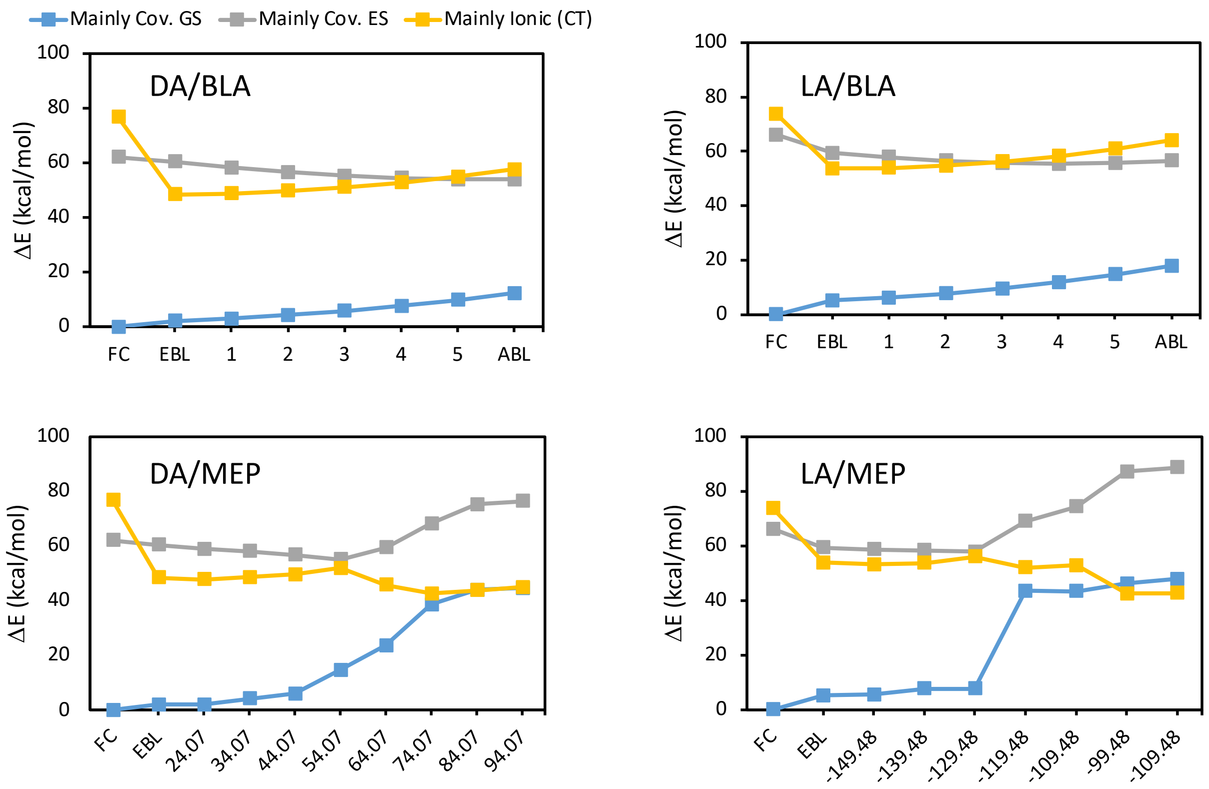

Supplementary Figure 8. Computed BLA and MEP. CASPT2//CASSCF/MM prediction of energy profiles for the BLA and minimum energy photoisomerization paths (MEPs) along the dihedral angle, C_{12}–C_{13}=C_{14}–C_{15}, for AT (LA) and 13C (DA) resting states. A very large S_1/S_2 mixing and significant energy barriers is observed along both paths, which are inconsistent with ultrafast and ballistic excited state decays. Source data are provided as a Source Data file.

As revealed from the figure, a very strong covalent/ionic (S_1 and S_2) mixing and significant energy barrier results into a slower photoisomerization process in both DA and LA states, inconsistent with experimental ballistic decay. It is worth noting that QM/MM relaxation of the

environment at the EBL point and keeping it fixed along the BLA MEP for the 13C system results in an even slightly larger barrier of 6.05 kcal mol^{-1}, thus reinforcing the result that the standard quadrupole model is unable to account for the observed photoinduced dynamical properties.

Also, semi-classical excited state dynamics on S_1 as shown in Supplementary Fig. 9 for both LA and DA states in *standard counterion* model corroborates the long living excited state population trapped in EBL minimum and hence, *non-ballistic* dynamics of this model. In other hand, the evolution of the S_1 energy of *protonated ASH212 OD2s* model relative to the ground state (S_0), clearly indicates that a CI seam is reached around 0.2 and 0.5 ps for DA and LA, respectively, which is compatible with the ballistic nature of the excited state decay in 13C, as well as sub-ps excited state dynamics observed in AT.

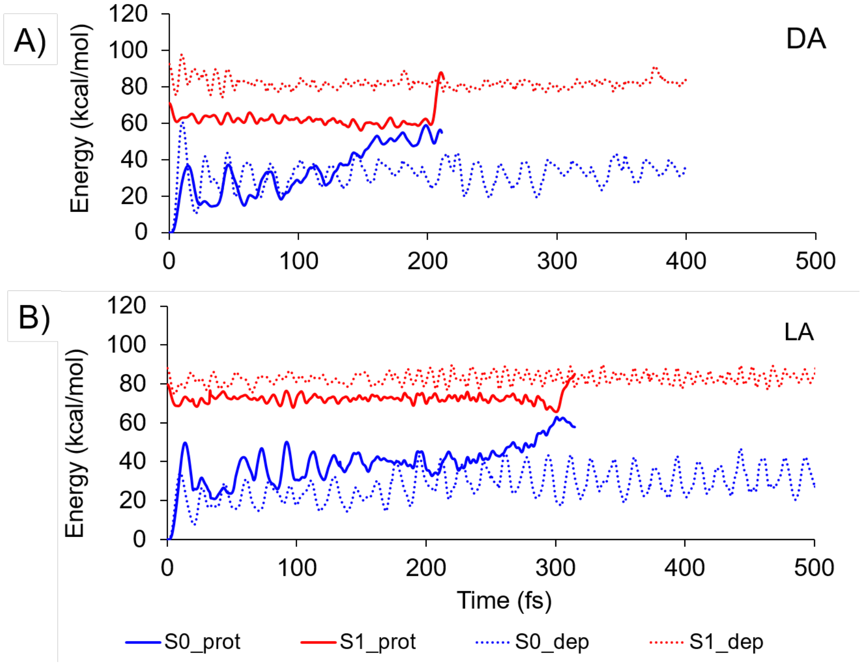

*Supplementary Figure 9. Semi-classical trajectories for DA and LA states. Semi-classical trajectories of rPSB photo-isomerization in **A)** DA and **B)** LA states of BR after population of the S_2 state with three-root state average in standard counterion complex (dashed lines) and after population of the S_1 state with two-root state average in protonated ASH212 OD2s (solid lines), at CASSCF/MM level of theory. The results corroborate the ballistic vs. non-ballistic dynamics of DA vs. LA **protonated counterion complex**, respectively. Source data are provided as a Source Data file.*

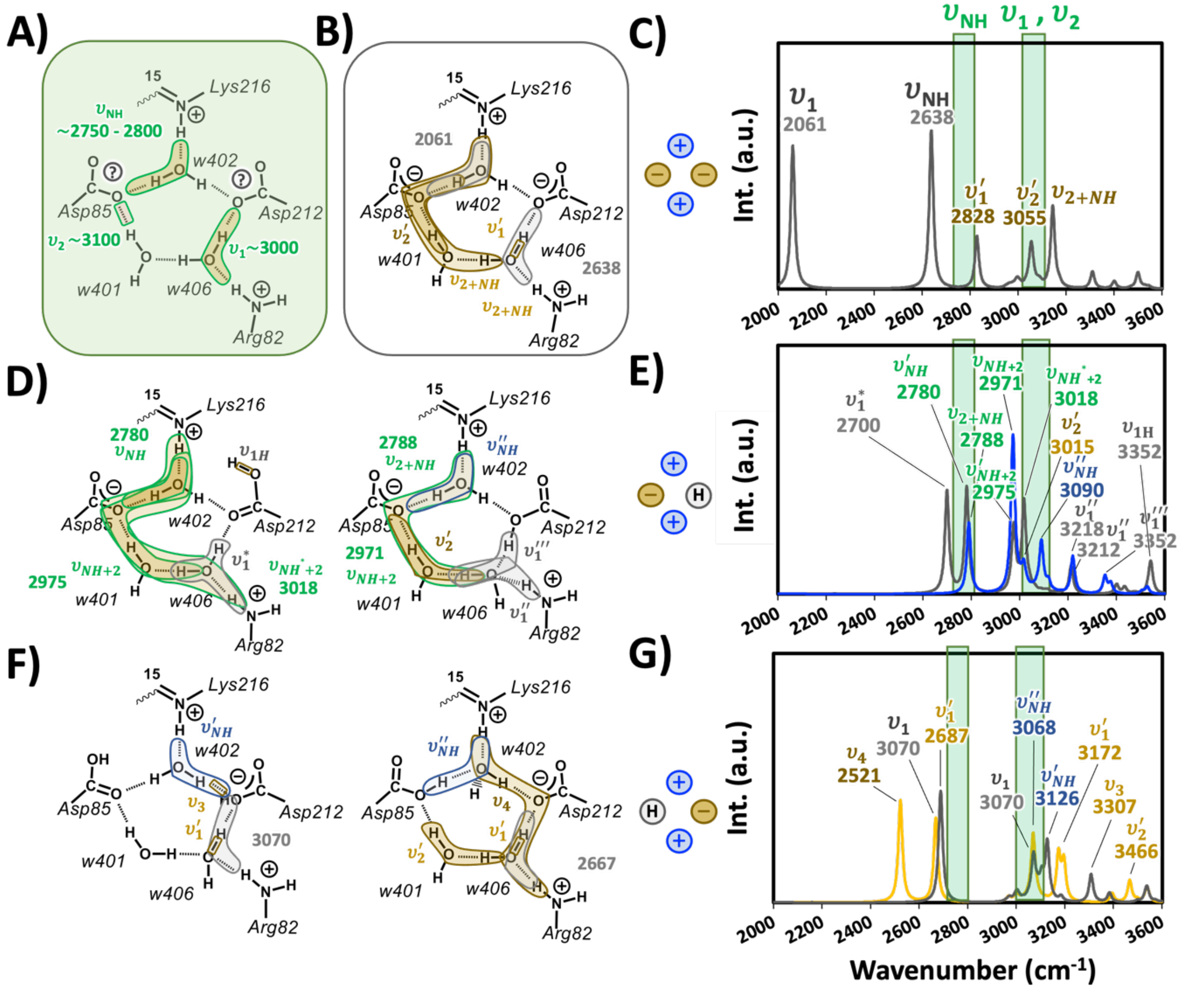

Supplementary Figure 10. The protonation state of the scrutinized complex counterion models and their IR frequencies. A) The main experimental vibrational frequencies associated to strong H-bonds in the standard model of the complex counterion, from ref. 8. B) The computed B3LYP/MM vibrational modes for the counterion with doubly deprotonated aspartates and C) the corresponding IR spectrum in the 2000-3600 cm^{-1} spectral range, including green bars to highlight the position of the experimental bands. The vibrational modes involved in the counterion model with D) protonated ASP212 and F) protonated ASP85, considering two examples of protonation sites on different carboxylate oxygens for each aspartate sidechain, and the corresponding IR spectra, E) and G), respectively. The (O–H, N–H) stretching associated to the IR peaks are sketched as coloured area in D) and F) are reported with label (and same colour scheme) in E) and G). IR frequencies are reported in cm^{-1}. Source data are provided as a Source Data file.

Supplementary Note 3: Photochemistry of K intermediate

3.1 640 nm pump pulse characterization

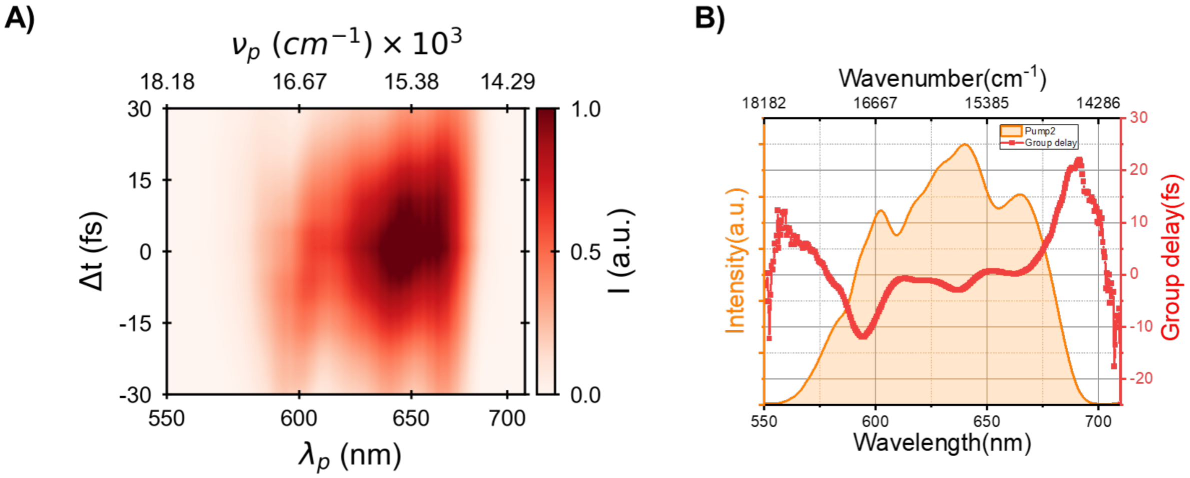

Supplementary Figure 11. Pump2 spectra. A) *2D colour map of FROG for the Pump2. X-axis represents wavelength, y-axis represents the delay and transmitted intensity is colour coded. B) The pump2 spectrum and group delay obtained from FROG. Left y-axis (orange) stands for normalized pump intensity and right y-axis(red) represents group delay. Source data are provided as a Source Data file.*

3.2 Quantification of K contribution in the three-pulse measurement

We have excited LA-BR or AT-BR with a 200 fs pulse center at 570 nm, which excited ~30% of the ground state population, ~60% of the excited population isomerize to K, and the rest returned to the ground state. Next, we introduced another 10-fs pulse covering 580-690 nm, 60 ps after the actinic excitation and carried out TA measurement. Hence our 3-pulse TA data contains AT and K, and with a suitable weighted factor of AT, we could isolate the K component. Similarly, we have carried out a 2-pulse measurement without actinic excitation, that is TA data of AT (Figure 5 in the main text).

We have carried out two 3-pulse TA measurement varying probe polarization with respect to pump polarization, (1) identical polarization of pumps and probe (VVV), and (2) orthogonal polarization between probe and pumps (VVH). The VVV TA spectrum at an early delay shows

positive absorption over 540 nm and negative absorption in the rest of the probe range with a characteristic stimulated emission minimum at 850 nm and an excited state absorption peak at 470 nm (Supplementary Fig. 12 B). The early TA spectrum of AT (2-pulse) alone and mixture (3-pulse) have similarities in the excited state absorption and stimulated emission region but a difference in the ground state bleach range, where the bleach range is red-shifted for the 3-pulse. However, at a longer 2-ps delay, 3-pulse data show a significant remainder of excited state absorption and stimulated emission. It is important to note that the excited state of AT almost disappears at 2-ps. Hence the excited state of K has a long-lived component. On the other hand, at 40 ps, the TA spectrum for VVV completely vanishes, indicating that the TA spectrum of K has precisely the opposite sign of AT. The TA spectra of VVH measurement also show the existence of long live decay component. Interestingly, at 40 ps delay, the TA spectrum of VVH measurement is nonzero, and it has an identical shape to AT TA spectrum (Supplementary Fig. 13). Thus, the AT and K ratio for the two measurements is different. To estimate the relative proportion of K in the two measurements, we compared the TA spectrum of VVV 3-pulse with VV 2-pulse at a 1.5 ps delay. Our estimate shows that the K component in VVV is 4.3 times more than VVH.

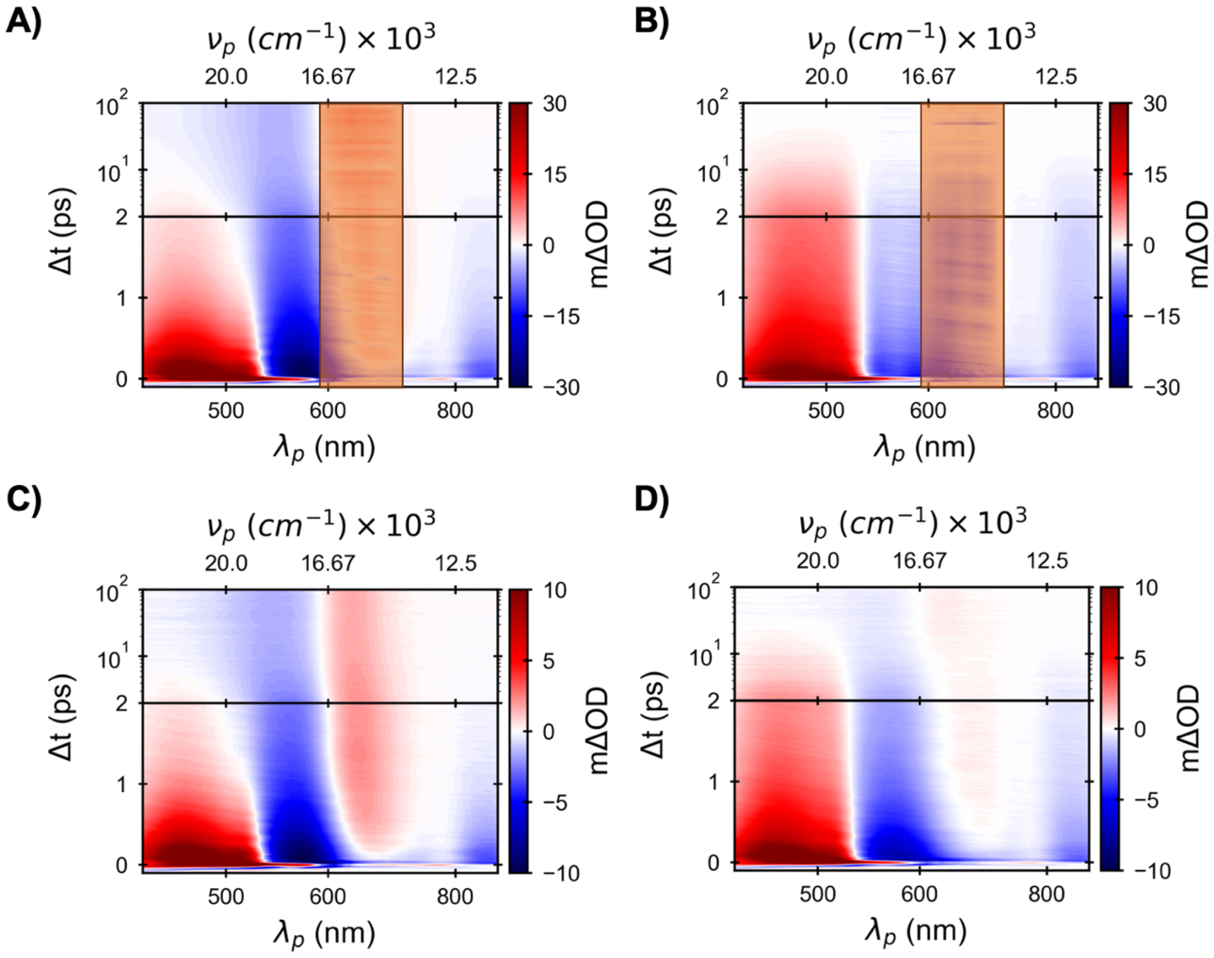

Supplementary Figure 12. TA data. A) The 2D colormap of TA data of AT-BR (VV) in the absence of actinic pulse. X-axis represents probe wavelength or wavenumber. Y-axis represents the delay between pump and probe. Initial 600 fs pump-probe delay is linear, and rest of the axis is logarithmic. The absorption difference is color coded, color bar at the right of the map presents the value of absorption difference. B) The 2D colormap of TA data of LA-BR (VVV) following the photoexcitation with actinic pulse. Pump2 excitation is used to obtain TA data. In addition, probe polarization is orthogonal to pump or pumps. C) is similar to A) except the probe polarization. D) is similar to B) except the probe polarization. For A) and B) probe polarization is identical to the pump polarization but in the case of C) and D) probe polarization is orthogonal to the pump polarization. Source data are provided as a Source Data file.

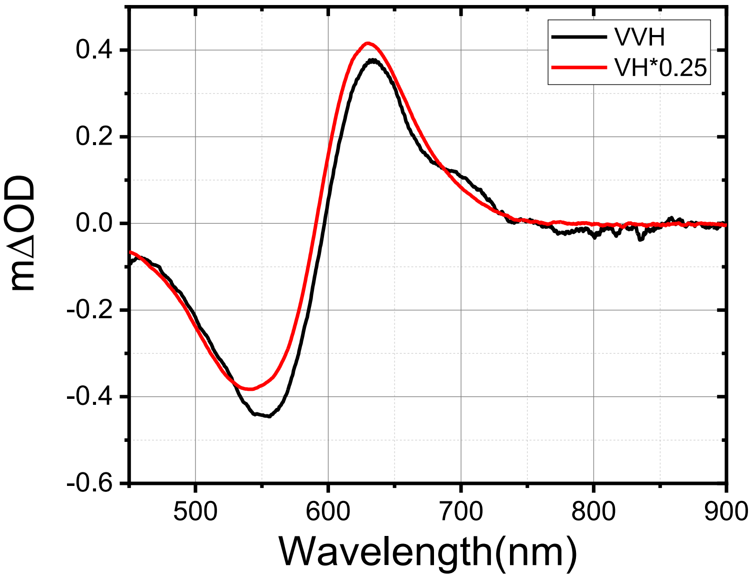

Supplementary Figure 13. TA spectra of VH and VVH at 100 ps. Source data are provided as a Source Data file.

Next, to quantify the weighted factor of AT in 3-pulse data, we have compared the amplitude of coherent modulation in TA spectra. Photoexcitation of AT with a 10-fs pulse creates coherent wave packet motion in the ground and excited-state potential surface. The Raman active modes of AT show a pronounced oscillatory modulation in the TA data. Around 520 nm probe wavelength, it exhibits a strong oscillatory component due to a ~200 cm^{-1} vibrational mode.[9] The 3-pulse data also has an identical modulation with half amplitude (Supplementary Fig. 14). We assume that the oscillatory component is only due to AT that cause the reduction of its amplitude in the 3-pulse measure. Thus, the factor ~0.5 of amplitude reflects the concentration of AT in the 3-pulse data. Similarly, a comparison of VH 2-pulse and 3-pulse VVH yields a factor of ~0.6. With this weighted factor, we have evaluated the K TA data. First, it is essential to note that the amplitude of K TA spectra obtained from the VVV scan is 4.3 times that VVH scan for all the delay, and it is consistent with our factor obtained from the comparison of raw data (Supplementary Fig. 15).

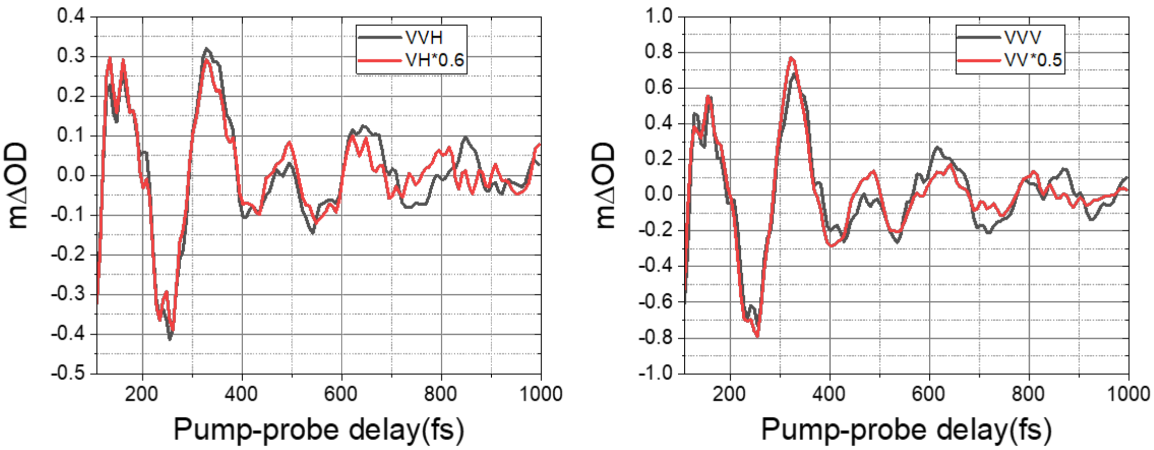

Supplementary Figure 14. Vibrionic modulation. Vibrionic modulation at 520 nm. Data for orthogonal pump and probe polarization (left). Data for identical pump and probe polarization (right). Source data are provided as a Source Data file.

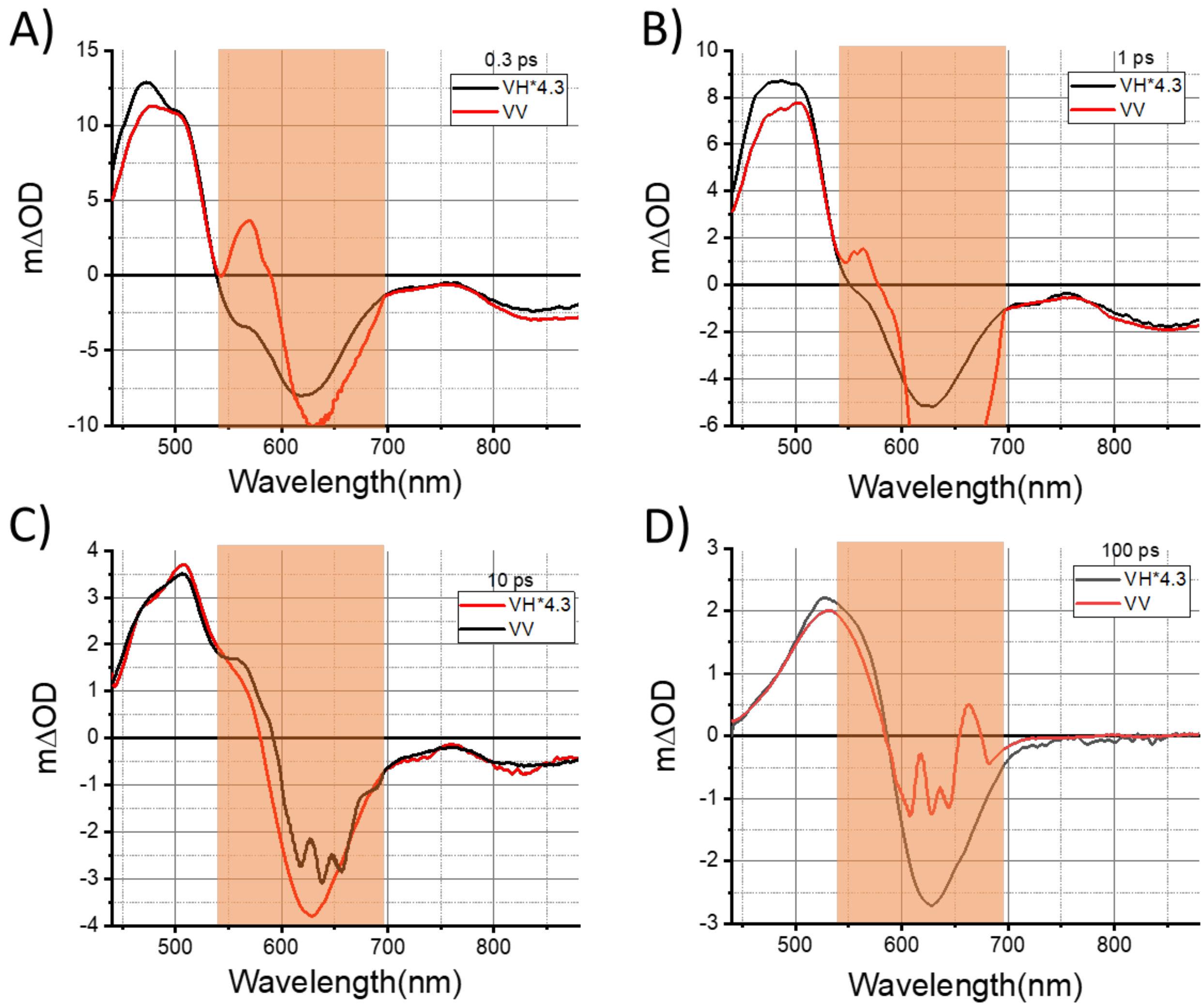

Supplementary Figure 15. TA spectra of K obtained from two orthogonal probe polarization. Pump scatter region in shaded. Two different probe polarizations show remarkable similarities over entire probe range. Source data are provided as a Source Data file.

3.3 Computational results

To explain the multiphase excited state decay of K state in more details, BLA and photoisomerization MEP profiles were obtained with the same QM/MM approach as was applied on the different conformations of ASP212 sidechain of LA and DA states. As shown in Supplementary Fig. 16, the computed $C_{13}=C_{14}$ photoisomerization MEPs, for different three conformers feature a barrierless pathway toward CI for OD1s and OD2s, and a 4.3 kcal mol^{-1} barrier for OD1a. This indicates that the C=C bond relaxation path (see Figure 7 in the main text) is the rate-determining step also for the K intermediate excited state decay. The results of C=C bond relaxation MEP for two different conformations of the carboxylic acid group of protonated ASP85 (computed for K intermediate state) exhibit always a barrierless FC → ABL pathway, demonstrating the fact that different conformations associated with the protonation of the ASP85 sidechain don't have any effect on the C=C bond relaxation MEPs (Supplementary Fig. 17).

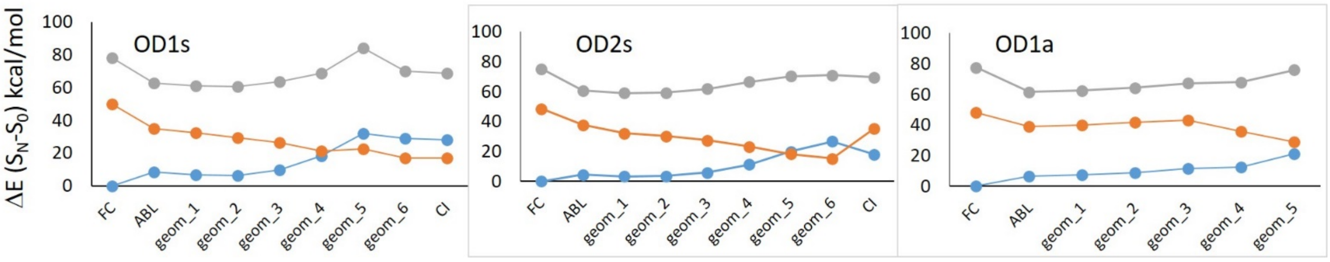

Supplementary Figure 16. Minimum energy paths (MEPs) in different protomers. Minimum energy paths (MEPs) along the C_{12}–C_{13}=C_{14}–C_{15} photoisomerization dihedral angle of the K intermediate state in different protomers, OD1s, OD2s, and OD1a at CASPT2//CASSCF/MM level of theory. Source data are provided as a Source Data file.

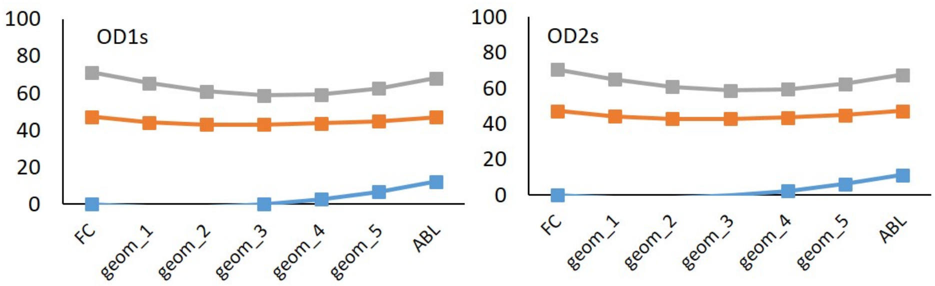

Supplementary Figure 17. Bond relaxation MEPs. The C=C bond relaxation MEPs (in kcal mol^{-1}) of FC $\rightarrow$ ABL pathway in two different conformations of the carboxylic acid group of protonated ASP85, in K intermediate state at CASPT2//CASSCF/MM level of theory. Source data are provided as a Source Data file.

Supplementary Table 2: Relative energies (in kcal mol^{-1}) of the different conformers, when ASP212 sidechain is protonated in all-trans (LA), 13-cis resting (DA) and K intermediate states of BR at CASPT2//CASSCF/MM level of theory.

Initial Conformer	all-*trans* BR	13-*cis* BR	K intermediate
OD2s	1.94	0.00	1.20
OD1a	0.00	Non-stable conformer	2.70
OD1s	9.33	13.93	0.00

Supplementary references

1. Weingart, O. *et al.* COBRAMM 2.0 — A software interface for tailoring molecular electronic structure calculations and running nanoscale (QM/MM) simulations. *J. Mol. Model.* **24**, 271 (2018).
2. Ernst, O. P. *et al.* Microbial and Animal Rhodopsins: Structures, Functions, and Molecular Mechanisms. *Chem. Rev.* **114**, 126–163 (2014).
3. Haupts, U., Tittor, J. & Oesterhelt, D. CLOSING IN ON BACTERIORHODOPSIN: Progress in Understanding the Molecule. *Annu. Rev. Biophys. Biomol. Struct.* **28**, 367–399 (1999).
4. Lanyi, J. K. Bacteriorhodopsin. *Annu. Rev. Physiol.* **66**, 665–688 (2004).
5. Aquilante, F. *et al.* Molcas 8: New capabilities for multiconfigurational quantum chemical calculations across the periodic table. *J. Comput. Chem.* **37**, 506–541 (2016).
6. Scherrer, P., Mathew, M. K., Sperling, W. & Stoeckenius, W. Retinal isomer ratio in dark-adapted purple membrane and bacteriorhodopsin monomers. *Biochemistry* **28**, 829–834 (1989).
7. Malakar, P. *et al.* Bidirectional Photochemistry of Antarctic Microbial Rhodopsin: Emerging Trend of Ballistic Photoisomerization from the 13- *cis* Resting State. *J. Phys. Chem. Lett.* **13**, 8134–8140 (2022).
8. Shibata, M., Yoshitsugu, M., Mizuide, N., Ihara, K. & Kandori, H. Halide Binding by the D212N Mutant of Bacteriorhodopsin Affects Hydrogen Bonding of Water in the Active Site. *Biochemistry* **46**, 7525–7535 (2007).
9. Kahan, A., Nahmias, O., Friedman, N., Sheves, M. & Ruhman, S. Following Photoinduced Dynamics in Bacteriorhodopsin with 7-fs Impulsive Vibrational Spectroscopy. *J. Am. Chem. Soc.* **129**, 537–546 (2007).
